# Detection of differentially methylated CpGs between tumour and adjacent benign cells in diagnostic prostate cancer samples

**DOI:** 10.1038/s41598-024-66488-x

**Published:** 2024-08-02

**Authors:** Liesel M. FitzGerald, Chol-hee Jung, Ee Ming Wong, JiHoon E. Joo, Julie K. Bassett, James G. Dowty, Xiaoyu Wang, James Y. Dai, Janet L. Stanford, Neil O’Callaghan, Tim Nottle, John Pedersen, Graham G. Giles, Melissa C. Southey

**Affiliations:** 1 Menzies Institute for Medical Research, University of Tasmania, Hobart, TAS Australia; 2 Melbourne Bioinformatics, University of Melbourne, Parkville, VIC Australia; 3 Precision Medicine, School of Clinical Sciences at Monash Health Medicine, Nursing and Health Sciences, Monash University, Clayton, VIC Australia; 4 Department of Clinical Pathology, The University of Melbourne, Parkville, VIC Australia; 5 Centre for Epidemiology and Biostatistics, School of Global and Population Health, University of Melbourne, Parkville, Australia; 6 Cancer Epidemiology Division, Cancer Council Victoria, Melbourne, VIC Australia; 7 Centre for Epidemiology and Biostatistics, Melbourne School of Population and Global Health, University of Melbourne, Parkville, VIC Australia; 8 Division of Public Health Sciences, Fred Hutchinson Cancer Center, Seattle, WA USA; 9 TissuPath, Mount Waverley, Melbourne, VIC Australia

**Keywords:** Prostate cancer, DNA methylation

## Abstract

Differentially methylated CpG sites (dmCpGs) that distinguish prostate tumour from adjacent benign tissue could aid in the diagnosis and prognosis of prostate cancer. Previously, the identification of such dmCpGs has only been undertaken in radical prostatectomy (RP) samples and not primary diagnostic tumour samples (needle biopsy or transurethral resection of the prostate). We interrogated an Australian dataset comprising 125 tumour and 43 adjacent histologically benign diagnostic tissue samples, including 41 paired samples, using the Infinium Human Methylation450 BeadChip. Regression analyses of paired tumour and adjacent benign samples identified 2,386 significant dmCpGs (Bonferroni *p* < 0.01; delta-β ≥ 40%), with LASSO regression selecting 16 dmCpGs that distinguished tumour samples in the full Australian diagnostic dataset (AUC = 0.99). Results were validated in independent North American (n_paired_ = 19; AUC = 0.87) and The Cancer Genome Atlas (TCGA; n_paired_ = 50; AUC = 0.94) RP datasets. Two of the 16 dmCpGs were in genes that were significantly down-regulated in Australian tumour samples (Bonferroni p < 0.01; *GSTM2* and *PRKCB*). Ten additional dmCpGs distinguished low (n = 34) and high Gleason (n = 88) score tumours in the diagnostic Australian dataset (AUC = 0.95), but these performed poorly when applied to the RP datasets (North American: AUC = 0.66; TCGA: AUC = 0.62). The DNA methylation marks identified here could augment and improve current diagnostic tests and/or form the basis of future prognostic tests.

## Introduction

The measurement of epigenetic markers, in particular DNA methylation of CpG sites (CpGs), is emerging as a promising and potentially cost-effective tool for cancer diagnosis and prognosis. Over the last decade, a transition from measuring individual CpGs in candidate genes to a more agnostic approach of assessing the wider methylome using array-based platforms has occurred. These platforms have evolved from focusing predominantly on promoter regions (e.g., the Illumina Infinium Human Methylation27 (HM27K) BeadChip) to including a considerable proportion of CpGs in gene body and intergenic regions (e.g., the Infinium HM450K and EPIC BeadChips).

Most prostate cancer studies to date have focused on identifying diagnostic markers, assessing DNA methylation in tumour and adjacent histologically benign tissue samples obtained from surgical prostatectomy^1–6^. Several differentially methylated genes have been identified and replicated, including *RARβ*^3,6,7^, *GSTP1*^3,6–9^, *AOX*1^3,4,10^ and *HIF3A*^1,3,6^. Although successful, these studies share a significant limitation: none was based on diagnostic tumour samples, such as those obtained from a needle biopsy or transurethral resection of the prostate (TURP). Instead, these studies utilised radical prostatectomy (RP) samples. Thus, it is unclear whether the markers and signatures that have been discovered in these RP studies are relevant to diagnostic tissue, especially in the earlier stages of tumour development.

Here, we applied the Infinium HM450K array to diagnostic prostate tissue samples, with an aim to identify differentially methylated CpGs (dmCpGs) that are directly relevant to diagnostic tissue. The relevance of these markers was then determined in unmatched diagnostic prostate tissue samples and in two independent sets of RP samples. Additionally, AmpliSeq™ transcriptome data, generated predominantly from the same diagnostic samples, allowed us to determine whether differential gene methylation altered gene expression. Finally, we investigated whether dmCpGs could distinguish Gleason score ≤ 7(3 + 4) (lower) from ≥ 7(4 + 3) (higher) tumours in the diagnostic setting and whether these were reproducible in RP samples.

## Results

Table 1 provides a summary of selected clinical characteristics of the diagnostic FFPE samples. Overall, a greater proportion of tumour samples was sourced from TRUS biopsies compared with benign samples (*p* = 0.02) and the study was enriched for more aggressive disease, including a higher proportion of cases who had died of prostate cancer.

**Table 1:** Study and clinical characteristics of prostate cancer participants.

Characteristics	Tumour (n = 122)	Adjacent benign (n = 42)	*p*-value^a^
Study n (%)			
MCCS	55 (45.1)	21 (50)	0.66
RFPCS	28 (23)	8 (19)	
RPR	12 (9.8)	5 (11.9)	
EOPCFS	25 (20.5)	6 (14.3)	
APC	2 (1.6)	2 (4.8)	
Age at diagnosis, years, n (%)			
< 65	56 (45.9)	15 (35.7)	0.25
≥ 65	66 (54.1)	27 (64.3)	
Sample type n (%)			
TRUS	69 (56.6)	15 (35.7)	0.02
TURP	53 (43.4)	27 (64.3)	
Gleason Score n (%)			
2–6	10 (8.2)		
7 = 3 + 4	24 (19.7)		
7 = 4 + 3	25 (20.5)		
8–10	63 (51.6)		
Vital Status n (%)			
Alive	28 (22.9)	12 (28.6)	0.82
Prostate cancer death	84 (68.9)	28 (66.6)	
Other death	9 (7.4)	2 (4.8)	
Unknown cause of death	1 (0.8)	0 (0.0)	

^a^*P*-values derived from the Chi-square test. A *p*-value < 0.05 is considered significant.

**Figure 1:**
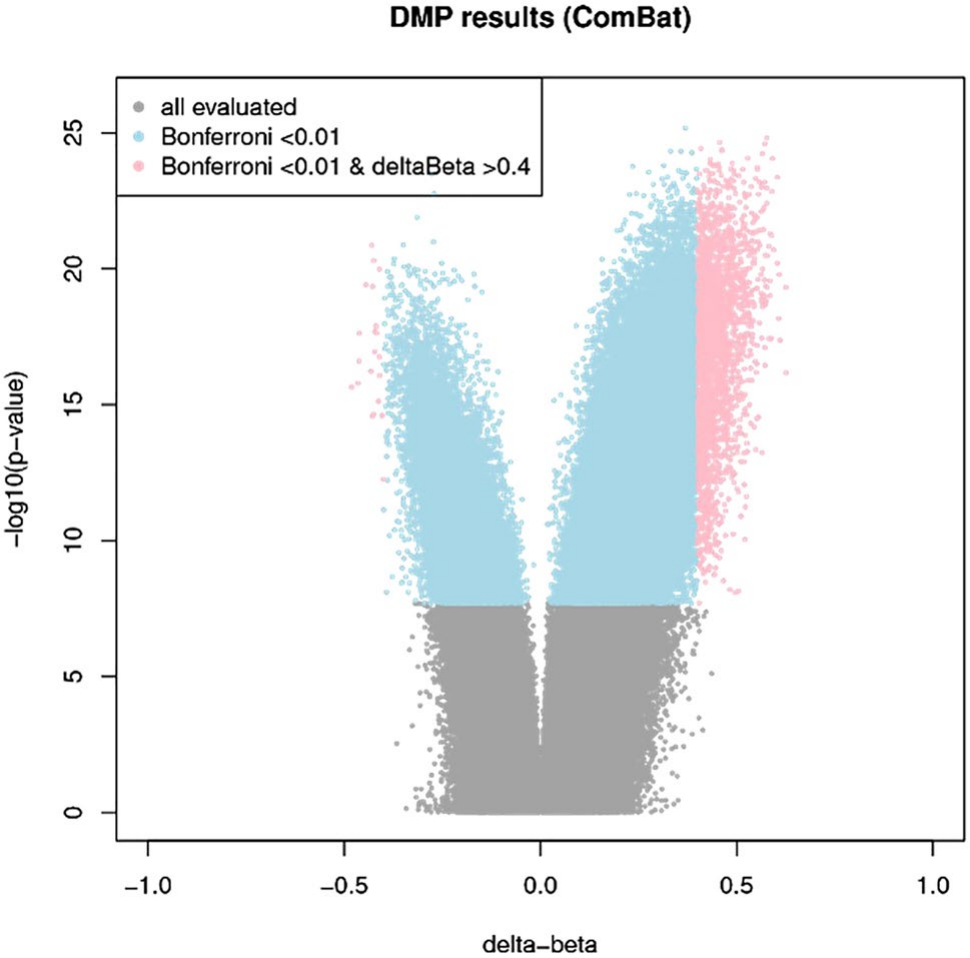
Volcano plot of DNA methylation in prostate tumour versus adjacent histologically benign tissue. Differentially methylated CpGs (dmCpGs) at Bonferroni corrected *p* < 0.01 are marked blue whilst dmCpGs at Bonferroni corrected *p* < 0.01 with a mean methylation difference of at least 40% between tumour and benign tissue are marked pink.

A total of 2386 CpGs were significantly differentially methylated between paired tumour and adjacent benign diagnostic samples (n = 39; Bonferroni *p* < 0.01, delta-β ≥ 40%; Fig. 1 and Supplementary Table S1). The majority were hypermethylated in tumour compared with adjacent benign samples (n = 2364; 99.1%). Hierarchical clustering analysis of all samples (n_tumour_ = 122; n_benign_ = 42) based on these 2386 dmCpGs resulted in the majority of tumour and adjacent benign samples clustering separately (Supplementary Figure S1). The distribution of dmCpGs in relation to all evaluated CpG sites by gene, CpG island and enhancer regions is provided in Supplementary Figure S2.

LASSO regression analysis was then run on our 2386 dmCpGs to determine the most representative set of jointly predictive markers that was still able to distinguish diagnostic tumour from benign samples. These analyses identified 16 dmCpGs that, as anticipated, successfully discriminated tumour and benign samples in our full dataset (including paired and unpaired samples: n_cancer_ = 122; n_benign_ = 42; AUC = 0.99; Table 2; Supplementary Figure S3). Of these 16 dmCpGs, 14 were hypermethylated whilst two, cg16709294 (*SFRS5*) and cg14179575 (*MC5R*), were hypomethylated in cancer compared with benign samples. In all instances except for cg06832339 (*STK31*), multiple significant dmCpGs were in close proximity to each of the LASSO-selected dmCpGs (Supplementary Figure S4). These 16 dmCpGs were also able to discriminate tumour from benign tissue in TCGA (n_paired_ = 50; AUC = 0.94) and FHCC RP datasets (n_paired_ = 19; AUC = 0.87; Supplementary Figure S3).

**Table 2:** Uncorrelated and significantly differentially methylated CpG sites between paired tumour and adjacent benign diagnostic samples**.**

CpG	Chr	Position	Gene	Gene Region	Bonferroni	deltaβ
cg11213690	7	149,112,402	Intergenic	Intergenic	2.92E−18	0.48
cg06832339	7	23,842,119	*STK31*	Body	4.19E−18	0.42
cg09414673	7	1,022,797	*CYP2W1*	TSS200	1.10E−17	0.45
cg05415131	11	58,940,866	*DTX4*	Body	5.06E−17	0.54
cg26310256	23	13,588,301	*EGFL6*	Body	1.09E−16	0.52
cg03217795	16	23,847,556	*PRKCB*	1st Exon	1.17E−16	0.42
cg03225210	7	143,579,951	*FAM115A*	5'UTR	1.79E−16	0.49
cg16670497	1	110,210,913	*GSTM2*	Body	2.52E−16	0.54
cg14621217	17	80,944,134	*B3GNTL1*	Body	6.20E−16	0.45
cg16709294	14	70,235,567	*SFRS5; LOC100289511*	Body; TSS1500	6.81E−16	0.43
cg21918559	23	36,976,052	Intergenic	Intergenic	9.36E−16	0.48
cg14775423	4	57,372,356	*ARL9*	5'UTR	1.74E−15	0.42
cg14179575	18	13,826,399	*MC5R*	1st Exon	2.43E−15	0.42
cg05057720	14	38,724,675	*CLEC14A*	1st Exon	4.26E−15	0.5
cg19713460	22	39,745,530	*SYNGR1*	TSS1500	6.48E−15	0.44
cg00927554	12	95,941,920	*USP44*	5'UTR	4.16E−11	0.41

Analysis of transcriptome data from 14 paired tumour and adjacent benign diagnostic samples identified 598 significantly differentially expressed genes (DEGs; Bonferroni < 0.01), 69.9% (n = 418) of which were down-regulated in tumour samples. Of our 16 LASSO-selected dmCpGs, two were present in genes, *PRKCB* and *GSTM2*, that were significantly down-regulated in tumour compared with benign samples (Bonferroni *p*-value < 0.01). When using an FDR significance threshold of < 0.01, *USP44* was also significantly down-regulated in tumour samples.

We then determined whether DNA methylation marks measured at diagnosis could distinguish samples based on Gleason score stratified into two categories – low ≤ 7(3 + 4) (n = 34) and high ≥ 7(4 + 3) (n = 88; Table 1). After accounting for multiple testing (FDR < 0.01), 1.799 CpGs were differentially methylated between low and high Gleason score cancer samples (Supplementary Table 2). However, no dmCpG achieved a delta-β ≥ 40%, instead the largest observed change was 21.2%. When applying a more conservative Bonferroni correction of < 0.01, the number of dmCpGs was reduced to 10 with the highest delta-β at 20% (Table 3). Whilst these 10 dmCpGs were able to successfully discriminate between low and high Gleason score tumours in our diagnostic dataset (AUC = 0.95; Supplemental Figure S), poor discrimination was achieved when applied to TCGA and FHCC RP samples (AUC = 0.62 and AUC = 0.66, respectively; Supplementary Figure S5).

**Table 3:** Significantly differentially methylated CpG sites between Gleason score ≤ 7(3 + 4) and ≥ 7(4 + 3) diagnostic tumour samples.

CpG	Chr	Position	Gene	Gene region	FDR	Bonferroni	deltaβ
cg05364411	17	37,183,206	Intergenic	Intergenic	0.00013	0.00013	0.06
cg12705693	5	912,860	*TRIP13*	Body	0.00050	0.00099	0.10
cg02245566	1	158,670,617	*OR6K2*	TSS200	0.00075	0.0028	0.20
cg19419246	19	45,950,425	Intergenic	Intergenic	0.00075	0.0032	0.09
cg27192635	7	149,749,941	Intergenic	Intergenic	0.00075	0.0042	0.13
cg03120102	16	78,991,723	*WWOX*	Body	0.00075	0.0051	0.08
cg22287731	11	55,066,025	Intergenic	Intergenic	0.00075	0.0055	0.15
cg21218627	9	132,999,496	*FREQ*	3'UTR	0.00075	0.0060	0.11
cg03751967	19	40,421,743	*FCGBP*	Body	0.00084	0.0076	0.12
cg01368068	7	78,368,660	*MAGI2*	Body	0.00084	0.0084	0.15

## Discussion

Previous studies have compared genome-wide DNA methylation in paired prostate tumour and adjacent benign tissue to identify markers with diagnostic utility, but none of these studies have used *diagnostic* samples (needle biopsy or transurethral resection of the prostate). Here, we assayed 39 pairs of diagnostic tumour-benign prostate tissue samples and identified 2,386 dmCpGs that distinguished tumour from benign samples. Further analysis reduced this number to a parsimonious set of 16 dmCpGs that were also able to distinguish tumour from benign samples in TCGA and FHCC RP datasets.

Our larger set of 2,386 dmCpGs included a number of genes that have previously been reported as differentially methylated in RP datasets^1,2,4^, including *GSTP1*^2–4,6–9,11^, *AOX1*^3,4,10^, *HIF3A*^1,3,4,6^, and *RARβ*^3,4,6,7,11^. After multiple testing correction, multiple CpGs at these loci were identified in our dataset as being significantly differentially methylated. When considering individual dmCpGs, our diagnostic study also replicated close to 95% of the 2,040 significantly dmCpGs identified in a North American RP study (based on FHCC samples)^3^, including 24 of their 27 most significant dmCpGs. While none of our 16 LASSO-selected dmCpGs overlapped with the 27 dmCpGs from the FHCC study, they were nonetheless able to clearly separate the majority of FHCC and TCGA tumour/benign sample pairs, demonstrating that dmCpGs identified in tissue obtained for diagnostic purposes can also be applied successfully to RP datasets. Whilst there is considerable overlap between our results and those from previous RP-based methylation studies, there is a proportion of dmCpGs that are more relevant in distinguishing tumour from benign tissue in diagnostic samples.

Of the 16 LASSO-selected dmCpGs, fourteen were located in or near genes, whereas two were annotated as being in intergenic regions; cg21918559 on the X chromosome (Xp21.1) and cg11213690 on chromosome 7 (7q36.1). Nine dmCpGs were present in genes with minimal prior evidence of a role in prostate cancer (Supplementary Table S3), while two dmCpGs, cg06832339 and cg09414673, are novel findings in respect to their ability to distinguish prostate tumour tissue. The remaining three dmCpGs, cg16670497, cg03217795 and cg00927554, were present in genes that were also differentially expressed in our dataset, *GSTM2*, *PRKCB* and *USP44*, respectively. Hypermethylation of *GSTM2* has been observed in several previous prostate cancer studies^3,11–13^, including reports that *GSTM2* hypermethylation could predict biochemical recurrence^12^ and that a three gene biomarker panel including *GSTM2* could provide a more accurate diagnosis^13^. Consistent with our findings, *Protein kinase C beta* (*PRKCB*; *PKCB*; 16p12.2-p12.1) was observed to be hypermethylated and downregulated in a Lithuanian RP dataset, and methylation of this gene was associated with biochemical relapse^14^. In 2019, Park et al*.* observed higher levels of *ubiquitin specific peptidase 44* gene (*USP44*; 12q22) expression in metastatic (PC3 and DU145) compared with benign or less invasive cell lines (RWPE1, RWPE2 and LNCaP)^15^. Knockdown experiments also suggested that USP44 promotes tumorigenic and cancer stem cell characteristics in PC3 and DU145 cells^15^. While these observations appear to contradict our findings, a more recent study reported *USP44* promoter methylation in plasma cell-free DNA of metastatic prostate cancer patients, which was significantly associated with worse overall survival^16^. Notably, our methylation and gene expression results were generated in cohorts enriched for high-risk primary tumours where ~ 72% and ~ 96% of samples had a Gleason score ≥ 7(4 + 3), respectively. Furthermore, studies of other cancers, including breast, colorectal, lung and clear cell renal cell carcinoma, have also found *USP44* to be hypermethylated^17^ and downregulated^17,18^, in addition to having an inhibitory effect on cell proliferation^19^.

Analyses based on Gleason score identified 10 significantly dmCpGs between low and high Gleason grade tumours. These dmCpGs performed poorly in discriminating low and high Gleason score tumours from the FHCC and TCGA RP datasets. This could be due to just over 70% of our samples being classified as high Gleason score, potentially limiting our statistical power to identify dmCpGs between the two groups. Furthermore, no Gleason score dmCpG achieved a methylation difference (delta-β value) of ≥ 40%, in fact the largest difference was only 21%. It is also difficult to compare our findings with those of previous studies, all of which were based on RP samples and presented limited data. While our study replicated three genes, *TCF7L1*^20^, *TERT*^11^ and *OPCML*^21^, none of these were in our 10 most significant Gleason score dmCpGs. Interestingly, Kron et al. (2009)^20^ identified several homeobox genes as differentially methylated between high and low Gleason score tumours; whilst we did not replicate these particular genes, four other homeobox genes, *HOXC12*, *HOXB9*, *HOXB4* and *HOXA3*, were highlighted, with 10 significantly dmCpGs present in the *HOXA3* gene. Overall, evidence from our and previous RP studies, strongly suggest dmCpGs may be able to distinguish low from high Gleason score tumours. However, studies to date have been constrained by small sample numbers, lack of consistency in how high and low Gleason score is defined, and limited replication. Thus, further investigation in both diagnostic and RP datasets is warranted, with systematic pathology reviews to ensure harmonisation of tumour grading.

Multiple studies have now demonstrated the utility of measuring DNA methylation in post-digital rectal examination urine samples^22–25^. Integrating non-invasive urinary methylation assays into standard clinical care has great potential to improve the specificity and sensitivity of initial diagnosis, in addition to being a critical tool in the monitoring of patients on active surveillance. These assays have also been shown to improve prognostication of prostate cancer^23,24^ and could thus provide vital information to inform treatment decisions.

Our study has considerable strengths. Foremost, it is the first study to identify dmCpGs that differentiate tumour from benign tissue in *diagnostic* prostate tumour samples. Through modifications to a HM450K protocol devised by our lab^26^, we were able to successfully assay diagnostic biopsies, in addition to TURP specimens. While there were significant differences between the clinical characteristics of these two diagnostic samples (Supplemental Table 3), as a proportion of men are diagnosed via TURP in a real-life clinical setting, it is important to include both sample types in biomarker discovery settings. Another strength of this study was the number of available paired tumour-benign samples. Here, we were able to analyse data from 39 paired samples whereas prior studies have assayed as little as four^4^ to 20 paired samples^3^, or in the case of Bjerre et al. (2019)^1^, no paired samples were included. Other strengths include the fact that all pathology specimens were reviewed and re-scored according to contemporary Gleason grading procedures at the time (2014; prior to the newer Grade Groups system), gene expression measures were undertaken in the same diagnostic sample cohort as the methylation measures and we had access to two independent datasets, FHCC and TCGA, for replication purposes. One of the major limitations of this study is that we were unable to replicate our findings in independent diagnostic tissue sample cohorts, as to the best of our knowledge, ours is the only such dataset. Another limitation was that due to a lack of clinical outcomes data, we were unable to investigate methylation signatures associated with clinically relevant features of disease (disease recurrence, metastasis or prostate-specific survival), other than tumour grade. It is also interesting to note that across our three study datasets, not all samples clustered according to the pathologists’ classification. Notably, of the three tumour samples that clustered unexpectedly in the study by Geybels et al*.*^3^, two also clustered unexpectedly when applying our markers. On closer inspection of the eight aberrant TCGA sample pairs, many paired samples clustered relatively closely together (Supplementary Figure S6). Unexpected clustering may be explained by contamination (i.e., presence of benign cells in tumour samples or vice versa), highlighting the importance of rigorous pathological review and dissection protocols. Gleason scores based on biopsy/TURP samples could also be considered a study limitation, given a proportion of cancers are upgraded upon surgery.

In summary, this genome-wide DNA methylation study of diagnostic prostate tumours has identified 16 dmCpGs that distinguish tumour from adjacent benign tissue in both diagnostic and RP samples. These markers should be further investigated to determine if they can be measured in urine and/or peripheral blood samples to augment and improve, or even replace, current invasive histopathological diagnostic methods. We further present a set of dmCpGs that distinguish diagnostic tumour samples of low- and high-grade disease. While these results were not replicated in two North American RP datasets, their association with tumour grade and other clinically relevant outcomes in additional diagnostic and RP datasets is warranted.

## Methods

### Study samples

Tumour material was sourced from five epidemiological studies led by the Cancer Council Victoria (CCV; see Supplementary Methods for full details).

### Study specimens

For all cases, formalin-fixed, paraffin-embedded (FFPE) diagnostic pathology material (a transrectal ultrasound (TRUS)-guided biopsy or transurethral section of the prostate (TURP)) was retrieved from the diagnostic service laboratory and reviewed by an experienced pathologist (JP; Table 1). Gleason score was reviewed and, if required, updated based on contemporary scoring practices in Australia at the time (2014). Information regarding a history of other cancer types was not available for this cohort.

This study included two groups of pathology specimens. After quality control (see Data Analysis below), the “paired dataset” comprised 39 specimens where separate areas of tumour and adjacent benign cells were identified for macrodissection. The “unpaired dataset” comprised 86 specimens after quality control, where only tumour (n = 83) or adjacent benign (n = 3) cells were identified for macrodissection.

### HM450K assays

DNA and RNA extraction from FFPE prostate tissues is described in the Supplemental Methods. The suitability of FFPE-derived DNA for application on the HM450K array was assessed using the workflow detailed in Wong et al*.* (2015)^26^ with minor modifications (see Supplementary Methods for details).

FFPE-derived DNA was bisulfite converted using the EZ DNA Methylation-Gold kit (Zymo, Irvine, CA, USA). Methylation was evaluated using the HM450K assay (Illumina, CA, USA) as per manufacturer’s instructions. FFPE-derived DNA extracted from paired tumour and benign cells were run adjacent to each other on the same HM450K array to control for inter-array variability.

### Independent HM450K datasets

HM450K data from two RP datasets, The Cancer Genome Atlas (TCGA) and a Fred Hutchinson Cancer Center (FHCC) study from North America, were available to compare findings from our diagnostic tissue-based analyses. HM450K data from 50 patients with matched primary tumour and benign (“solid tissue normal”) were downloaded from TCGA data portal (https://tcga-data.nci.gove/tcga/). The FHCC resource consisted of 19 matched tumour-benign samples (described in Geybels et al.^3^) and a dataset of 461 unmatched tumour samples from Caucasian men (described in Geybels et al.^27^). Gleason score information was available for both TCGA and FHCC datasets.

### Transcriptome data

Fourteen tumour samples with matched benign tissue (including 12 samples from the methylation dataset) had gene expression data available. These data were generated using the AmpliSeq™ Transcriptome Human Gene Expression Kit (AmpliSeq assay; ThermoFisher Scientific), as described in FitzGerald et al*.* (2018)^28^ and in the Supplemental Methods.

### Data analysis

The Chi-square test was used to determine whether selected study and clinical features of PrCa differed between tumour and adjacent benign samples. A *p*-value of < 0.05 was considered significant.

HM450K array data were imported into the R environment (R Programming Software version 3.4) and processed using the *Minfi* package version 1.24.0^29^. Samples with a CpG detection rate < 95% were removed (n = 0) and CpGs designated as a SNP (n = 65) or with a detection rate of *p* ≤ 0.01 in less than 80% of the samples (n = 1074) were removed, resulting in 483,167 CpGs for subsequent analyses. The data were then normalised using SWAN^30^ and batch effects were removed using ComBat^31^.

Methylation β- and M-values were calculated and principal component analysis (PCA) performed to identify sample outliers. Logistic regression and least absolute shrinkage and selection operator (LASSO) regression analyses were performed to identify dmCpGs between paired tumour and benign samples as described in detail in the Supplemental Methods.

Logistic regression analysis was used to identify dmCpGs between tumours with a lower or higher Gleason score. Low Gleason score was defined as ≤ 7(3 + 4) and high as Gleason score ≥ 7(4 + 3). dmCpGs with an FDR < 0.01 in tumour samples were considered significant, but to reduce the number of markers, we also considered a more conservative Bonferroni *p*-value of < 0.01.

*edgeR*^32^ was used for read-count normalisation and to determine differential gene expression (DGE) between the 14 tumour and benign sample pairs (Bonferroni *p*-value < 0.01) in the top differentially methylated genes. DGE between Gleason score groups could not be determined due to the majority (96%) of samples with gene expression data having Gleason score ≥ 7(4 + 3).

To evaluate the discrimination performance of the dmCpGs identified above, receiver-operator characteristic (ROC) curve analysis was performed in the full diagnostic dataset and two independent RP datasets using coefficients associated with the dmCpGs from the diagnostic paired dataset.

### Ethics approval

Ethics approval for this study was obtained from the Human Research Ethics Committee Cancer Council Victoria, Australia (H1306). Written informed consent was gained for all participants. The Fred Hutchinson Cancer Center study was approved by the Institutional Review Board and informed consent was obtained from all study participants.

### Supplementary Information

Supplementary Information.

Supplementary Tables.

## Data Availability

LMF and CHJ had full access to all the data in the study and take responsibility for the integrity of the data and the accuracy of the data analysis. The data underlying this study are available in the article and its online supplementary material, or from the corresponding author or MCS upon reasonable request for ethically approved research.
