# Supplementary material for: Detection of differentially methylated CpGs between tumour and adjacent benign cells in diagnostic prostate cancer samples
Source: Sci Rep. 2024 Aug 2;14:17877. doi: 10.1038/s41598-024-66488-x (PMC11297152; doi:10.1038/s41598-024-66488-x)
Supplement: Supplementary file 1 — Supplementary Information. [file 41598_2024_66488_MOESM1_ESM.pdf]

Supplementary Methods

Study samples

Cases from all five studies including the Melbourne Collaborative Cohort Study (MCCS)[1,2]; the Risk Factors For Prostate Cancer Case–Control Study (RFPCS)[3,4]; the Early Onset Prostate Cancer Family Study (EOPCFS)[5]; the Radical Prostatectomy Registry (RPR)[6] and the Aggressive Prostate Cancer (APC) study[7], were followed passively from date of diagnosis via linkage to the Victorian Registry of Births, Deaths, and Marriages, and the National Death Index, both of which obtained cause of death data from the Australian Bureau of Statistics. Follow-up for this study ended 31 March 2013, which was the latest date for which complete cause of death information was available at the time of specimen selection. Written informed consent was obtained from each participant and the study was approved by the Human Research Ethics Committee of the Cancer Council Victoria.

Nucleic acid extraction from FFPE prostate tumour material

Tumour areas representative of the overall Gleason score and adjacent benign areas were identified by a pathologist (JP & TN) and marked directly on the representative H&E-stained section. On average two 3µm unstained sections were deparaffinized using a standard xylene and ethanol procedure prior to macrodissection. DNA was extracted using the QIAamp DNA FFPE Kit (Qiagen, Hilden, Germany) with modifications to the protocol as outlined in Wong *et al.* (2015)[8] and stored at -20°C. DNA was measured using the Qubit® dsDNA BR Assay kit on the Qubit® Fluorometer (Life Technologies, CA, USA) as per manufacturer's instructions. For RNA extraction, on average, five 8µm (freshly cut) unstained sections were deparaffinized using a standard xylene and ethanol procedure prior to macrodissection. RNA was extracted using the RecoverAll™ Total Nucleic Acid Isolation Kit for FFPE (ThermoFisher Scientific) as per the manufacturer's instructions except that RNA was eluted in a final volume of 30µl of nuclease-free water and stored at −80°C. The quantity and quality of FFPE RNA was measured using the Agilent 2100 Bioanalyzer system (RNA 6000 Pico Kit) according to the manufacturer's instructions.

HM450K assessment workflow

The suitability of FFPE-derived DNA for application on the HM450K array was assessed using the workflow detailed in Wong et al. (2015)[8] with minor modifications. Due to limited amounts of DNA obtained from prostate FFPE tissue, QC checkpoint 2 was omitted in order to preserve as much DNA as possible for sodium bisulfite conversion and the [illegible]Cq at QC checkpoint 3 was lowered to ≥2 compared with the negative control. Where either tumour or benign FFPE-derived DNA did not pass any of the QC checkpoints, its paired FFPE-derived DNA was not progressed further in the workflow.

Data analysis

Methylation β-values were calculated (intensity of methylated allele/[intensity of unmethylated allele + intensity of methylated allele + 100]) and ranged from 0 (unmethylated) to 1 (100% methylated)[9]. Methylation M-values were defined as the logit transformation of the β-values. Principal component analysis (PCA) was performed to examine the clustering of tumour and benign samples and to identify outliers. Four samples were removed due to incorrect clustering, including three cancer samples and one adjacent benign sample. Logistic regression analysis, run within the limma package (version 3.34.9), was used to identify dmCpGs between paired tumour and benign samples. To correct for multiple testing and select dmCpGs with potential biological relevance, a Bonferroni p-value <0.01 and a delta-β value of ≥0.4 (i.e., a ≥40% difference) was considered statistically significant. Least absolute shrinkage and selection operator (LASSO) regression analysis was then performed using the glmnet package (version 2.0.18) to identify and remove any potentially correlated markers and reduce the number of dmCpGs to a minimal set that is still able to distinguish cancer and benign samples. Unlike dmCpG identification, LASSO regression was not restricted to paired samples.

cDNA generation, library preparation and transcriptome assays

Complementary DNA (cDNA) was generated with SuperScript® VILO™ cDNA Synthesis Kit using 40–110ng of FFPE RNA or 4ng of lymphoblastic cell line-derived RNA. Barcoded libraries were generated

(18-24 target cycles) using Ion AmpliSeq technology and the human gene expression core panel. Libraries were quantified by qPCR using the Ion Library Quantification Kit, diluted to 100pM and pooled equally, with six individual samples per pool. Each chip contained at least one lymphoblastic cell line-derived RNA sample as a positive control and a sample that was either replicated on the same chip or within the same batch.

References

1. Geurts YM, Dugué PA, Joo JE*, et al.* (2018). Novel associations between blood DNA methylation and body mass index in middle-aged and older adults. *Int J Obes (Lond)* 42, 887-96.

2. Giles GG, and English DR. (2002). The Melbourne Collaborative Cohort Study. *IARC Sci Publ* 156, 69-70.

3. Giles GG, Severi G, McCredie MR*, et al.* (2001). Smoking and prostate cancer: findings from an Australian case-control study. *Ann Oncol* 12, 761-5.

4. Severi G, Hayes VM, Padilla EJ*, et al.* (2007). The common variant rs1447295 on chromosome 8q24 and prostate cancer risk: results from an Australian population-based case-control study. *Cancer Epidemiol Biomarkers Prev* 16, 610-2.

5. Eeles RA, Kote-Jarai Z, Al Olama AA*, et al.* (2009). Identification of seven new prostate cancer susceptibility loci through a genome-wide association study. *Nat Genet* 41, 1116-21.

6. Bolton D, Severi G, Millar JL*, et al.* (2009). A whole of population-based series of radical prostatectomy in Victoria, 1995 to 2000. *Aust N Z J Public Health* 33, 527-33.

7. FitzGerald LM, Zhao S, Leonardson A*, et al.* (2018). Germline variants in IL4, MGMT and AKT1 are associated with prostate cancer-specific mortality: An analysis of 12,082 prostate cancer cases. *Prostate Cancer Prostatic Dis* 21, 228-37.

8. Wong EM, Joo JE, McLean CA*, et al.* (2015). Tools for translational epigenetic studies involving formalin-fixed paraffin-embedded human tissue: applying the Infinium HumanMethyation450 Beadchip assay to large population-based studies. *BMC Res Notes* 8, 543.

9. Du P, Zhang X, Huang CC*, et al.* (2010). Comparison of Beta-value and M-value methods for quantifying methylation levels by microarray analysis. *BMC Bioinformatics* 11, 587.

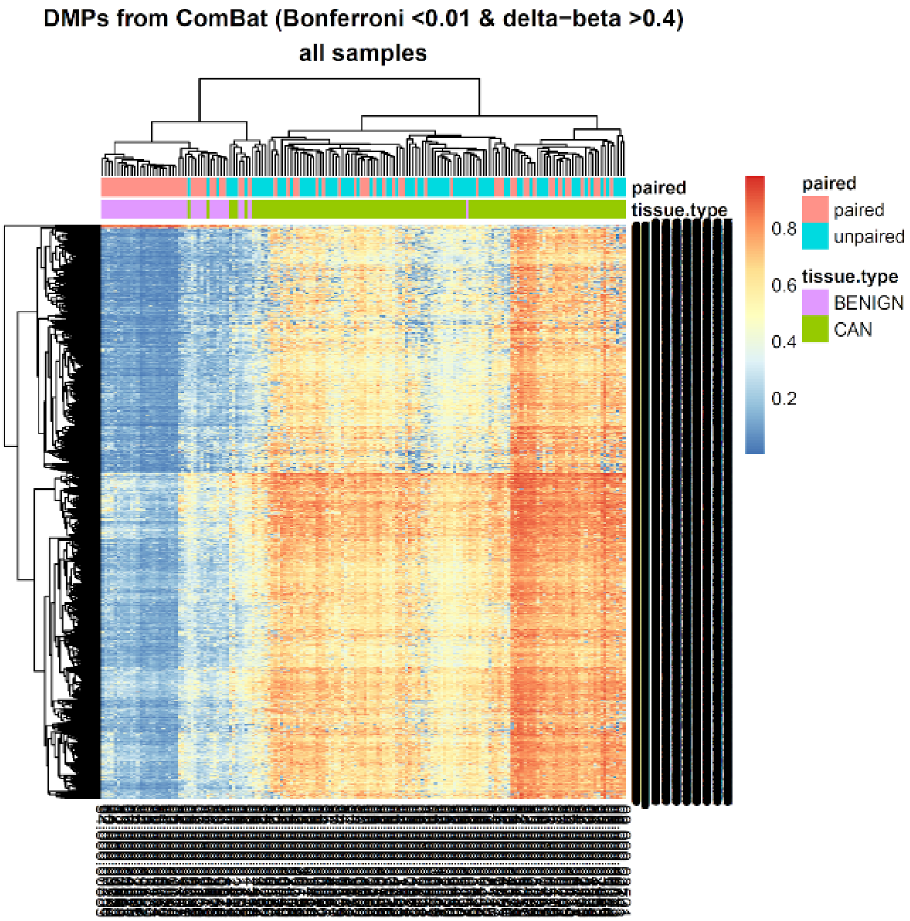

Supplementary Figure S1. Unsupervised hierarchical clustering of all tumour (n=122) and adjacent benign (n=42) samples based on the 2,386 significant dmCpGs (Bonferroni p<0.01; delta-β ≥40%).

A.

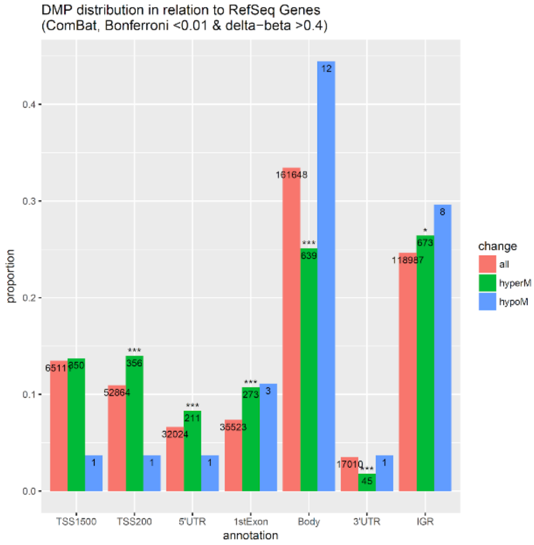

B.

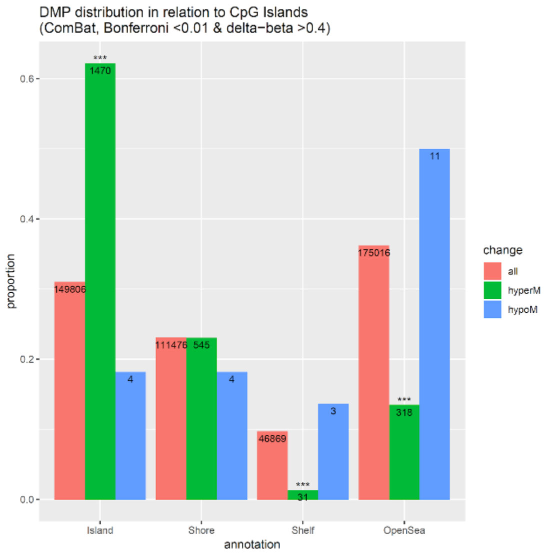

C.

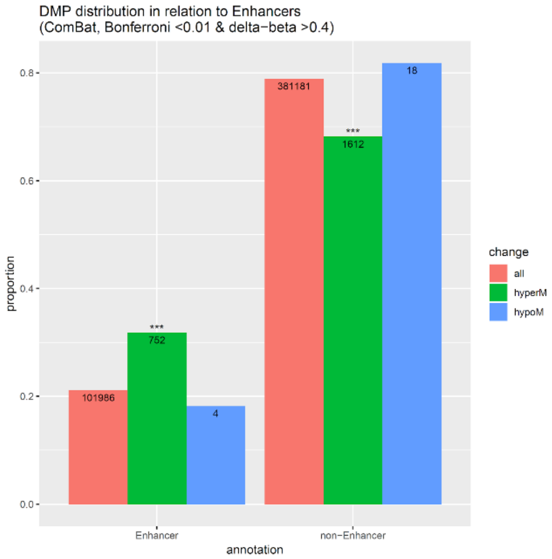

Supplementary Figure S2. The frequencies of all evaluated CpG sites compared to those that were significantly differentially hyper- (n=2,364) or hypo-methylated (n=22) by: **A.** Gene region; **B.** Location relative to CpG islands; and **C.** Enhancer region. All regions are based on Illumina HM450 methylation data. Statistically significant differences are marked as: *p-value* <0.05 (*); <0.01 (**); <0.001 (***).

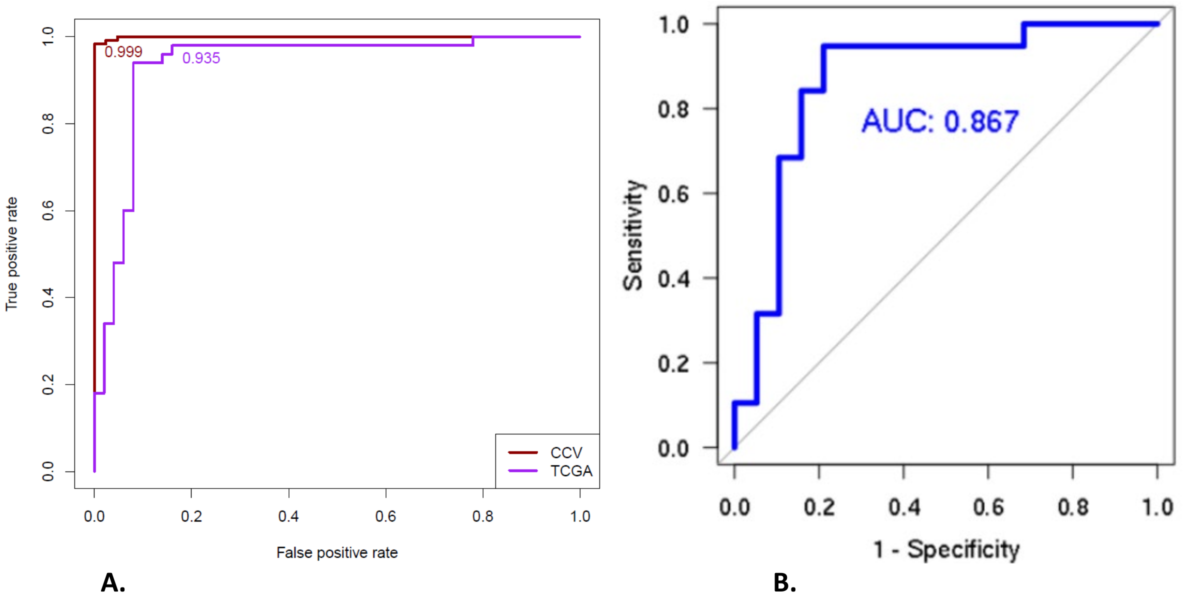

Supplementary Figure S3. Receiver operating characteristic curves and AUC values for 16 dmCpGs separating tumour and adjacent benign prostate samples from **A.** the diagnostic CCV dataset (Brown line; AUC = 0.999) and the radical prostatectomy TCGA dataset (Purple line; AUC = 0.935) and **B.** the FHCC dataset (AUC = 0.867).

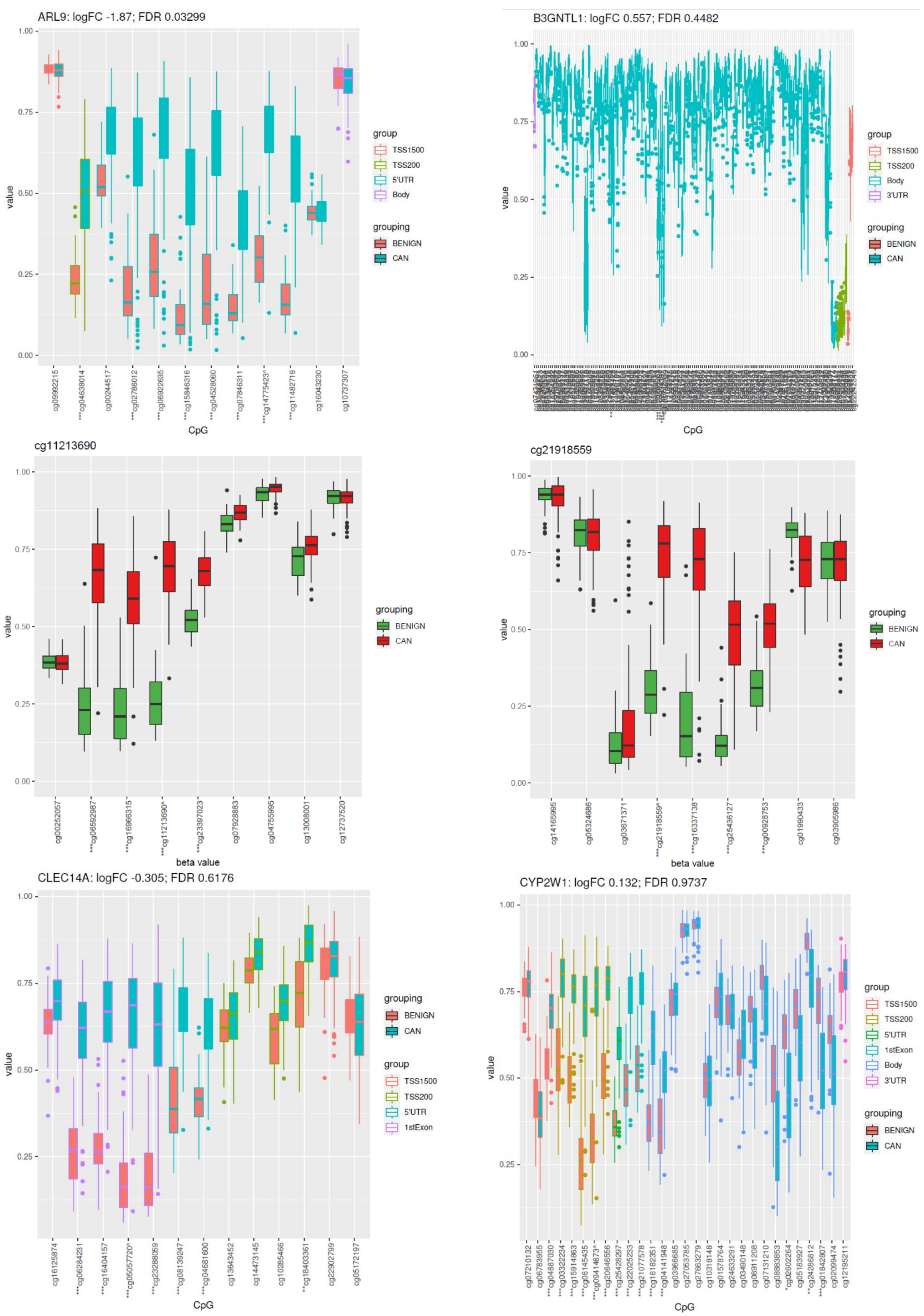

Supplementary Figure S4: Methylation marks surrounding each of the 16 dmCpGs that successfully distinguish diagnostic tumour and benign samples. The LASSO-selected dmCpGs are indicated with a ^, e.g., cg16709294^. Significant dmCpGs are indicated with * (p=0.01), ** (p=0.001) or *** (p=0.0001).

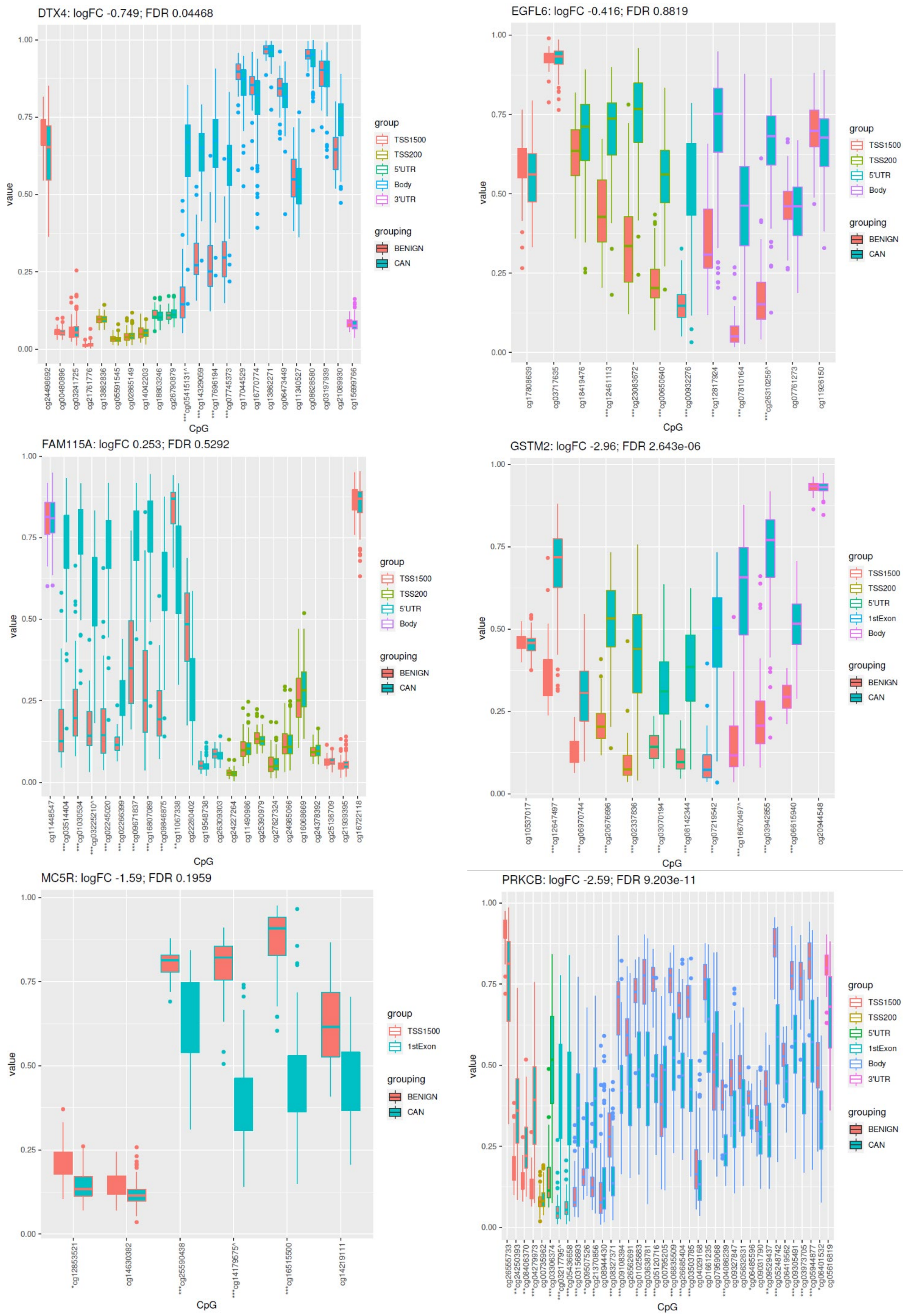

Supplementary Figure S4: cont.

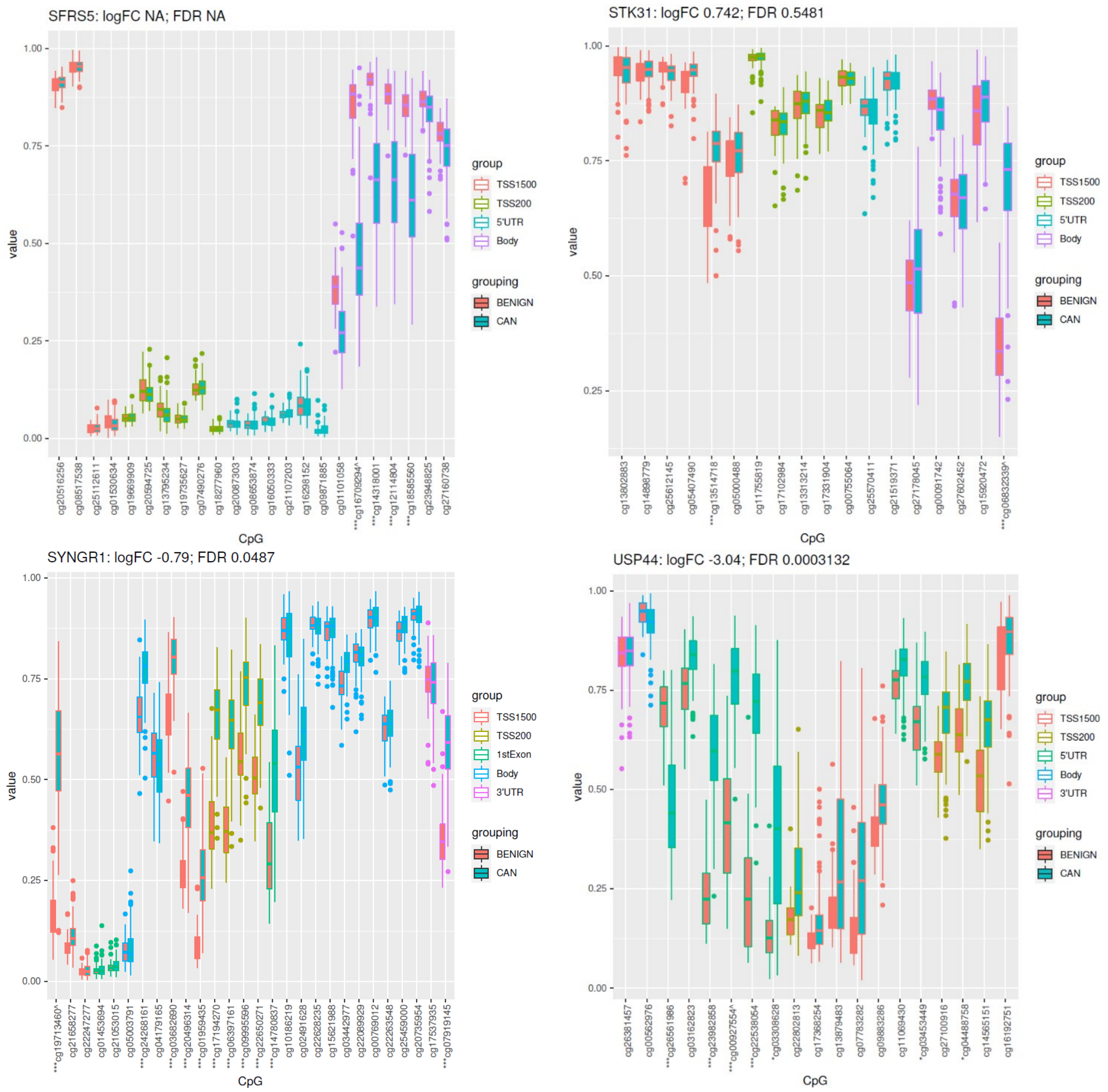

Supplementary Figure S4: cont.

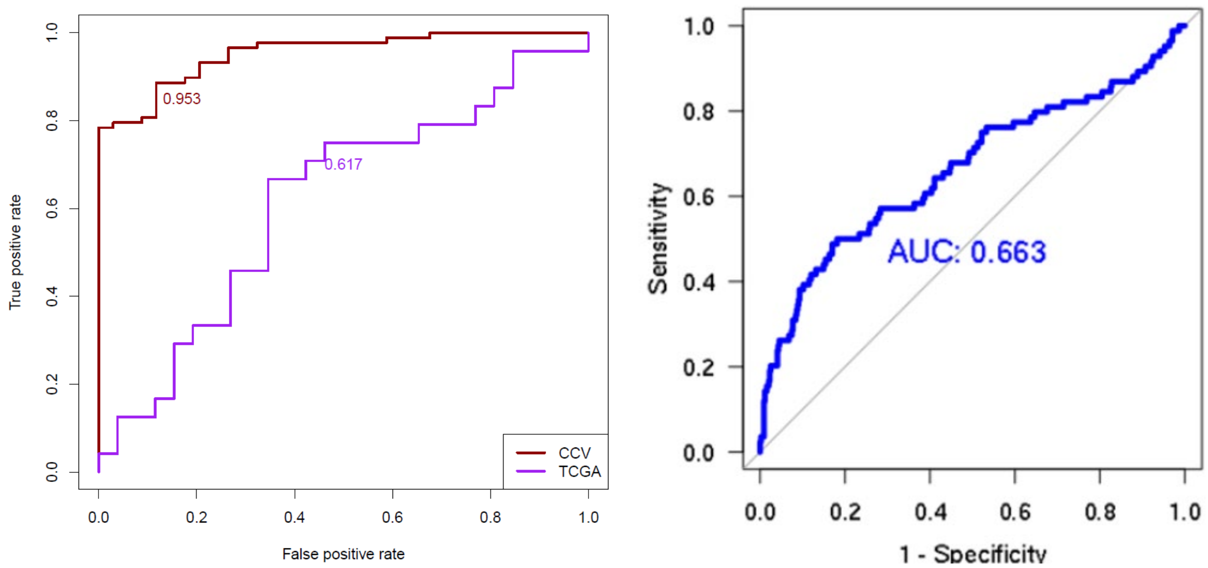

A. **B.**

Figure S5. Receiver operating characteristic curves and AUC values for 10 dmCpGs separating low Gleason score (≤7(3+4)) and high Gleason score (≥7(4+3)) samples from **A.** the diagnostic CCV dataset (Brown line; AUC = 0.953) and the radical prostatectomy TCGA dataset (Purple line; AUC = 0.617) and **B.** the FHCC dataset (AUC = 0.663).

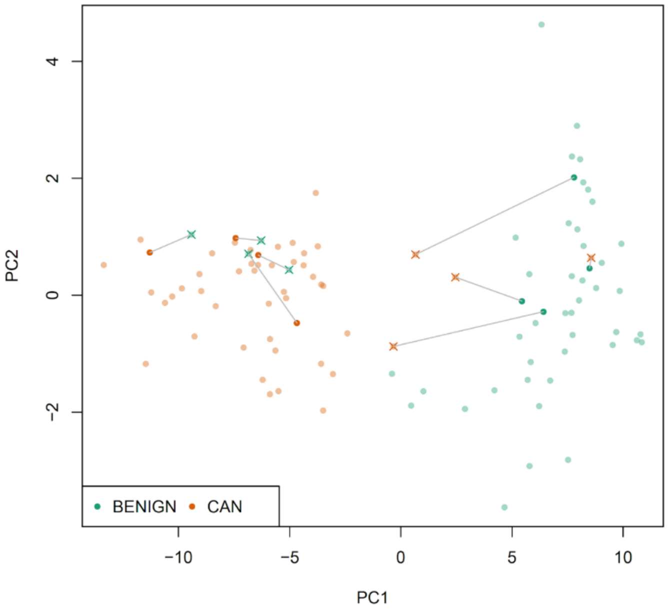

Supplementary Figure S6. Unsupervised principal component analysis of the most highly ranked 16 dmCpGs in TCGA samples. Misclustered samples are marked with an X and their matched sample is linked with a grey line. Cancer samples are marked orange and benign samples are marked green.
